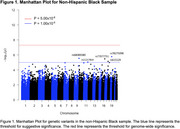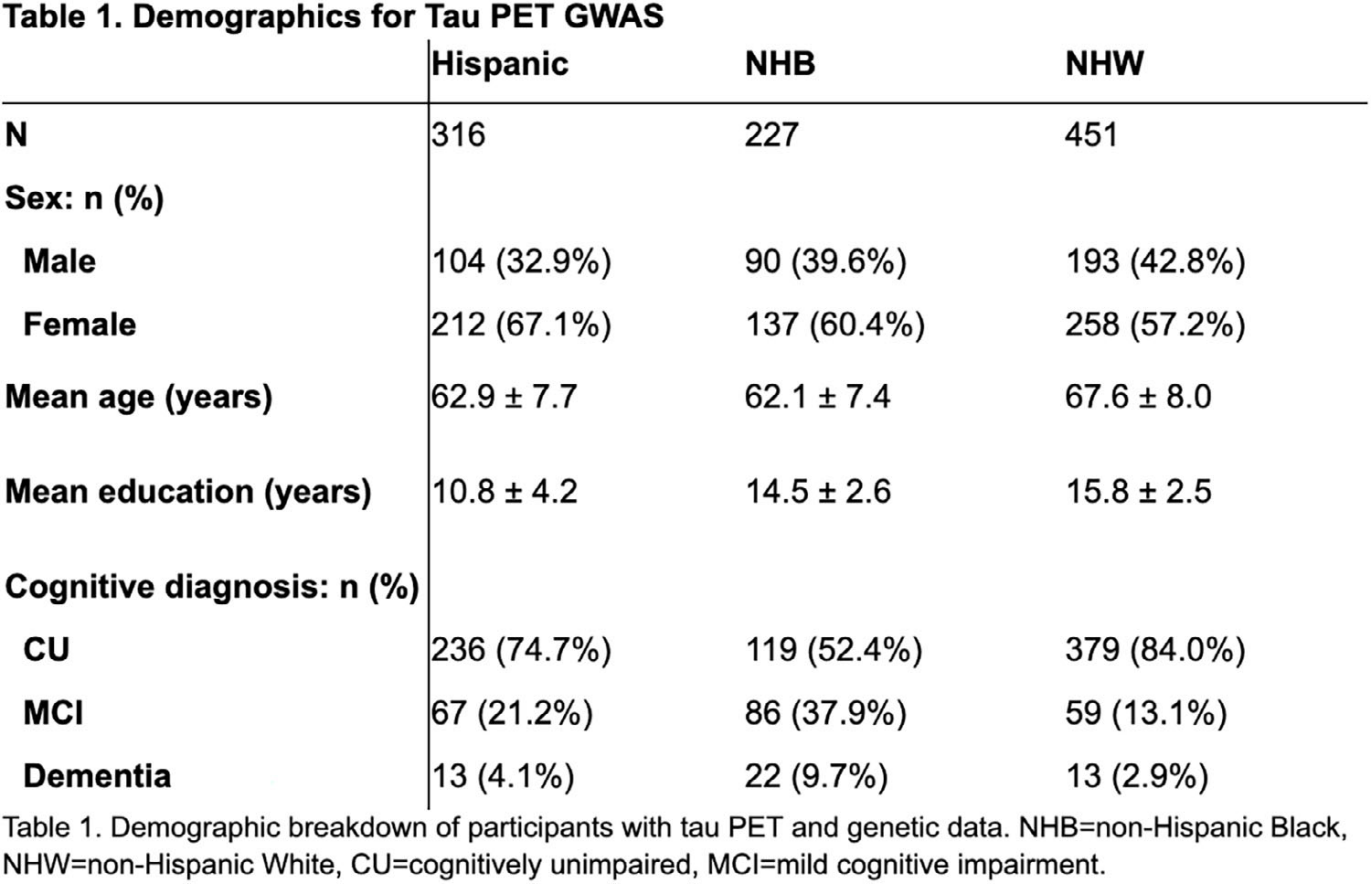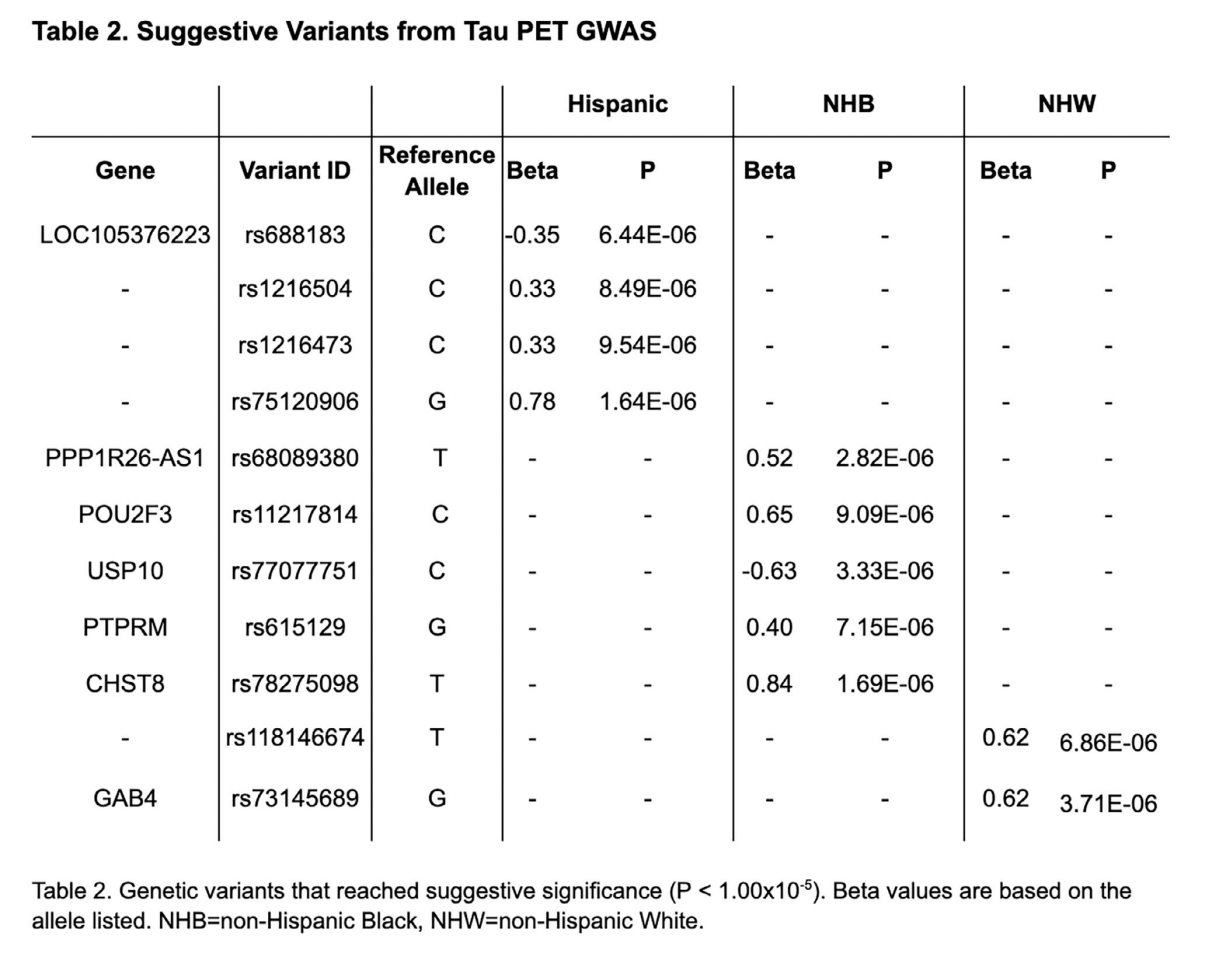# Genetic Variants and Brain Tau Levels

**DOI:** 10.1002/alz70855_105772

**Published:** 2025-12-23

**Authors:** Danielle Luu, Koral V Wheeler, Maxwell W Hand, Noelle Lee, Arthur W. Toga, Sid E. O'Bryant, Kristine Yaffe, Robert Barber, Nicole Phillips, Meredith N. Braskie

**Affiliations:** ^1^ Imaging Genetics Center, Mark and Mary Stevens Neuroimaging and Informatics Institute, Keck School of Medicine, University of Southern California, Marina del Rey, CA, USA; ^2^ University of North Texas Health Science Center, Fort Worth, TX, USA; ^3^ University of California, San Francisco, Weill Institute for Neurosciences, San Francisco, CA, USA

## Abstract

**Background:**

Genetic variants influence Alzheimer's Disease (AD) risk and development. However, it is unclear which genes specifically influence AD neuropathology like tau accumulation, especially across ethnoracial groups. We performed a genome‐wide association study (GWAS) across three ethnoracial groups to identify variants related to brain tau positron emission tomography (PET) signal in the medial temporal lobe (MTL).

**Methods:**

We observed 994 participants (mean age 64.8 ± 8.2; 734 cognitively unimpaired (CU), 212 mild cognitively impaired (MCI), 48 with dementia) from the Health and Aging Brain Study‐Health Disparities (HABS‐HD), with available self‐reported ethnoracial identity (316 Hispanic, 227 non‐Hispanic Black (NHB), and 451 non‐Hispanic White (NHW)) (Table 1). PI‐2620 PET (Siemens BioGraph Vision 450 PET/CT) was acquired and standardized uptake value ratios (SUVRs) were calculated in the MTL using inferior cerebellar gray matter as the reference region. Genetic data underwent standard quality control for GWAS, and principal component analysis was performed separately within each ethnoracial group to account for population structure. Using PLINK, we performed association tests in each ethnoracial group covarying for age, sex, cognitive diagnosis, education, and the first two principal components. We defined genome‐wide significance as *p* < 5.00x10^‐8^ and suggestive significance as *p* < 1.00x10^‐5^ based on previous literature.

**Results:**

Eleven variants reached suggestive significance (*p* < 1.00x10^‐5^) across ethnoracial groups (Table 2). These included SNPs in the NHB sample (Figure 1) in genes associated with hippocampal volume and function (*USP10*), metabolic regulation (*USP10, CHST8*), lipids and apolipoproteins (*PTPRM*), and inflammation (*USP10*), all of which have been implicated in AD risk. Separate variants in the Hispanic and NHW samples were identified but not related to known AD factors.

**Conclusion:**

We identified genetic variants potentially associated with MTL tau accumulation in NHB participants, located in genes having prior associations with AD risk factors. We will assess these relationships in larger samples when they become available in HABS‐HD. If confirmed, these results will enhance our understanding of dementia risk across ethnoracial groups and guide future research into personalized medicine and precision healthcare.